# Association of American Medical Editors

**Published:** 1875-04-15

**Authors:** 


### ASSOCIATION OF AMERICAN MEDICAL EDITORS.

THE members of this association, and all others connected editorially with the regular Medical Press of this country, are requested to meet in the parlors of the Galt House, in Louisville, Ky., at 7:30 o'clock, on Monday evening, May 3d, 1875; being the evening previous to the assembling of the American Medical Association. The Association of Medical Editors was formed in New Orleans, in May, 1869, for the cultivation of friendly relations, consultations concerning topics of mutual interest, and the urging of concert of action in the advancement of education, both preliminary and medical.

The proposition for its formation was made by Dr. Theophilus Parvin, of Indianapolis, whose object was to make it the instrument of accomplishing much good. Up to the present time, however, the number of editors present at the several meetings has been so limited as to render it impracticable to carry into execution the original plans of the Association. We hope there will be a larger number of members present at the coming meeting. It is the duty of the President to deliver to the Association an address, and we doubt not the present incumbent of that office, Dr. W. S. Edgar, of St. Louis, will be prepared to discharge that duty on the evening named.

